# Lung function reductions associated with motor vehicle density in chronic obstructive pulmonary disease: a cross-sectional study

**DOI:** 10.1186/s12931-016-0451-3

**Published:** 2016-10-24

**Authors:** Monika Nitschke, Sarah L. Appleton, Qiaoyu Li, Graeme R. Tucker, Pushan Shah, Peng Bi, Dino L. Pisaniello, Robert J. Adams

**Affiliations:** 1 Department for Health and Ageing, 11 Hindmarsh Square, Adelaide, South Australia Australia; 2 The Health Observatory, Discipline of Medicine, The University of Adelaide, Adelaide, South Australia Australia; 3 The School of Public Health, University of Adelaide, Adelaide, South Australia Australia; 4 Environment Protection Authority, Adelaide, South Australia Australia

**Keywords:** COPD, Vehicle density, Lung function, Air pollution, Cross-sectional study

## Abstract

**Background:**

Motor vehicle-related air pollution can potentially impair lung function. The effect of pollution in people with compromised pulmonary function such as in COPD has not been previously investigated. To examine the association of lung function with motor vehicle density in people with spirometrically determined COPD in a cross-sectional study.

**Methods:**

In 2004–06, The North West Adelaide Health Study (NWAHS), a biomedical cohort of adults assessed pre and post-bronchodilator spirometry (*n* = 3,103). Traffic density, obtained from the motor vehicle inventory maintained by the South Australian Environment Protection Authority, was expressed as the daily numbers of vehicles travelling within a 200 m diameter zone around participants’ geocoded residences.

**Results:**

In subjects with COPD (FEV_1_/FVC <0.7, *n* = 221, 7.1 %), increasing daily vehicle density was associated with statistically significant decreases in lung function parameters after adjustment for smoking and socio-economic variables. Mean (95 % CI) post-bronchodilator % predicted FEV_1_ was 81 % (76–87) in the low (≤7179/day) compared with 71 % (67–75) in the high (≥15,270/day) vehicle exposure group (*p* < 0.05). Linear regression analysis in all subjects with COPD showed significant decrements in post-bronchodilator FEV_1_/FVC ratio and % predicted FEV_1_ of 0.03 and 0.05 % respectively per daily increase in 1000 vehicles. In men with COPD (*n* = 150), the corresponding reductions were 0.03 and 0.06 %. Smaller, non-significant decrements were seen in females. No difference was seen in those without COPD.

**Conclusions:**

Vehicle traffic density was associated with significant reductions in lung function in people with COPD. Urban planning should consider the health impacts for those with pre-existing respiratory conditions.

**Electronic supplementary material:**

The online version of this article (doi:10.1186/s12931-016-0451-3) contains supplementary material, which is available to authorized users.

## Background

The prevalence of chronic obstructive pulmonary disease (COPD) ranges from 5 to 9 % in those aged over 40 years, with an increase in prevalence predicted in some countries [1, 2]. Based on the global disability-adjusted life-year (DALY) estimates, COPD has been ranked eighth out of 20 of the most common diseases in 2012 [3]. Although mortality rates of COPD are decreasing in Australia, mainly due to reduced smoking, it still represents about 4 % of all deaths in the population aged 55 and over and the economic burden of this disease is substantial [1].

While it is agreed that cigarette smoking is a major cause of COPD, and about 80 % is estimated to be attributable to smoking, there is also evidence for other risk factors [2].

Biologically-based impacts of particulate matter (PM) on the lungs have been shown in a range of studies that have explored the response of the immune system to particulates leading to exacerbations of symptoms and airflow restriction in people with COPD [4]. It has been hypothesised that over time these PM-stimulated inflammatory processes could lead to airway remodelling contributing to the aetiological pathway to COPD [4–7]. COPD is a progressive inflammatory disease and the additional effect of exposure to particulate matter and gases may add to the already ongoing inflammatory action and airflow obstruction which may have been originally caused by cigarette smoking or occupational exposures.

There is some evidence from three recent cross-sectional general population studies that lung function decline is associated with PM with an aerodynamic diameter of less than 10 μm (PM10) and proxy indicators of traffic in adults [8–10]. A recent review concluded that there is suggestive evidence of adverse lung function effects associated with increasing traffic-related exposure in the general population of children and adults, but the available evidence precluded a causal relationship [11]. A commensurate investigation among the sub-population of people with COPD has not yet been undertaken. Considering that people with COPD have to preserve remaining lung function, it is important to find out the relevance of where people live, their exposure to traffic and the potential effects on their lung function.

The aim of this study was to examine effects on lung function of vehicular traffic density measured around the homes of adults with COPD using data from an ongoing cohort study conducted in Adelaide, South Australia (SA).

## Methods

### Study design and population

This study associates lung function data with traffic density using a cross-sectional design.

Participants with COPD were identified by post-bronchodilator spirometry conducted at stage 2 (May 2004–February 2006) of the North West Adelaide Health Study (NWAHS), a representative biomedical population cohort study of adults aged 18 years or older randomly selected from the Electronic White Pages telephone directory and living in the north-western suburbs of Adelaide, South Australia (regional population, 0.6 million of 1.2 million). The majority of the participants (96 %) were of European decent. The methods of the NWAHS and the validity of these methods of selection to achieve an unbiased sample at Stage 1 (biomedical examination *n* = 4060, 69 % response rate) have been described previously [12].

Stage 2 of the NWAHS utilised identical methodology to stage 1 and clinic data were obtained on 79 % (*n* = 3206) of the stage 1 NWAHS population. Smoking status, and socio-economic determinants were identified by computer assisted telephone interview. Spirometry and anthropometry were conducted with standardised reproducible methodology according to the 1987 ATS criteria [12].

Approval for the conduct of the study was obtained from the Ethics of Human Research Committees of the University of Adelaide and the Central Northern Adelaide Health Service, and all subjects gave written consent. This particular sub-study was approved by the NWAHS study team and the University of Adelaide Human Research Ethics Committee.

### Pulmonary function measurements

Spirometry was conducted using a Microlab 3300 Spirometer (Micro Medical LTD, Kent, United Kingdom). Each subject performed at least three acceptable and reproducible forced vital capacity manoeuvres where the largest FVC (and FEV_1_) and second largest FVC (and FEV_1_) from acceptable manoeuvres did not vary by more than 100 ml and 5 % of the largest volume. Data are presented as post-bronchodilator percentage of predicted forced expiratory volume in 1 second (FEV_1_) and forced vital capacity (FVC). COPD was considered present if the post bronchodilator FEV_1_: FVC ratio was <0.7 given recent findings in relation to COPD pathology and adverse outcomes in people classified by the fixed ratio, but not by the lower limit of normal [13–17]. Percentage reversibility of airways obstruction was determined as post-bronchodilator FEV_1_-pre-bronchodilator FEV_1_/pre-bronchodilator FEV_1_. Pre-bronchodilator data can be viewed in the supplement.

### Traffic density

Density of vehicles within a certain buffer is a commonly used traffic indicator relating to health outcomes, and is highly correlated to measurements of nitrogen dioxide and PM2.5, the main contenders for exposures that affect respiratory health [18].

Proxy traffic exposure in the form of vehicle counts on all road sections surrounding the geocode of participants within a circle of 200 m diameter was calculated using ArcGIS 9.3 [19]. A buffer of 200 m around each home was selected based on studies that indicated that traffic-related exposure is receding to background levels within 200 to 300 m from major roads [11;18]. Proximity to major roads in meters (>10 000 cars per day), a traffic metric of lower exposure quality, was included into Table 1 (characteristics of participants) to assess distribution of proximity categories between people with COPD and non-COPD.

**Table 1:** Characteristics of the NWHAS population overall and by 24 h vehicle density in relation to COPD

	COPD				No COPD				Total
characteristics	Low *N* = 61 27.6 %	Med *N* = 67 30.3 %	High *N* = 93 42.1 %	Total *N* = 221 100 %	Low *N* = 791 27.5 %	Med *N* = 873 30.3 %	High *N* = 1,218 42.3 %	Total *N* = 2, 882 100 %	(3,103)
Mean (SD)									
Age (years)*	68.2 (11.9)				53.9 (15.3)				%
BMI	26.7 (4.9)	27.0 (3.8)	27.6 (5.8)	27.2 (5.0)	28.4 (5.6)	28.2 (5.5)	28.2 (5.5)	28.2 (5.6)	28.2 (5.5)
Gender*									
male female	65.6 34.4	77.6 22.4	62.4 37.6	67.9 32.1	45.1 54.9	45.7 54.3	47.5 52.6	46.3 53.7	46.9 53.1
Age (category)									
20–29 years	–	1.5	–	0.5	5.3	4.2	4.8	4.8	4.5
30–39 years	1.6	1.5	–	0.9	13.3	14.9	13.3	13.8	12.9
40–49 years	8.2	7.5	4.3	6.3	24.2	22.2	21.8	22.6	21.4
50–59 years	16.4	10.5	18.3	15.4	24.8	22.0	24.3	23.7	23.1
60–69 years	29.5	31.4	21.5	26.7	16.7	19.1	16.8	15.5	18.1
70 years and over	44.3	47.8	55.9	50.2	15.8	17.5	19.0	17.7	20.0
Current smoking*	27.9	18.0	23.7	23.1	18.0	16.1	17.3	17.0	17.6
Ever smoked*	49.2	65.7	52.7	55.7	32.2	36.5	33.0	33.9	32.6
Education level*									
Left school at ≤15	35.0	29.9	35.6	33.6	19.2	20.9	21.0	20.5	21.5
After 15	25.0	16.4	21.7	21.7	31.7	28.6	27.2	28.9	28.4
Trade/apprentice	15.0	22.4	21.1	19.8	14.9	14.9	12.2	13.8	14.2
Certificate/Diploma	21.7	28.4	14.4	20.7	22.4	22.0	24.0	23.0	22.8
Bachelor degree	3.3	3.0	5.6	4.2	11.9	13.6	15.5	14.0	13.3
Household Income*									
≤ $20, 000	29.6	43.8	41.4	39.0	25.0	25.8	27.0	26.1	27.0
> $20, 000 − 40, 000	51.9	32.8	39.1	40.5	25.8	24.1	25.3	25.1	26.1
> $40, 000 − 60, 000	7.4	14.1	12.6	11.7	20.7	21.2	20.8	20.9	20.3
> $60, 000	11.1	9.4	9.4	8.8	28.5	28.9	26.9	27.9	26.6
SEIFA IRSD quintiles									
Lowest-low quintile combined	27.5	27.5	45.0	59.6	28.0	28.1	44.0	58.2	58.3
Middle quintile	23.1	33.3	43.6	17.7	19.0	31.8	49.2	16.6	16.7
High and highest quintile combined	32.0	34.0	34.0	22.7	31.7	34.7	33.6	25.2	25.0
Proximity to major road									
> 300 m	77.1	84.6	35.5	61.5	79.0	77.7	25.1	55.8	55.7
150–300 m	23.0	16.4	18.3	19.0	21.0	21.9	21.7	21.6	21.6
< 150 m	0	0	46.2	19.5	0	0.5	53.2	22.6	22.7

*p < 0.05 for the overall comparison of COPD versus no COPD group

High ≥15,270 vehicles, medium 7180–15269 and low density group ≤7179 vehicles per 24 h

*SEIFA* socio-economic indexes for areas, relative socio-economic advantage and disadvantage (IRSAD)

Additional file 1: Figure S1 in the supplement shows an example of a location of one of the participants with surrounding road links. The traffic count is based on the sum of traffic travelling in both directions over a full year, passing a roadside observation point, divided by the number of days in the year. The motor vehicle inventory maintained by the SA Environment Protection Authority (EPA) includes annual average daily traffic counts of traffic for all road links in a GIS compatible database. A large part of the counts are based on traffic surveys, while counts on local road links (approximately up to one third) are modelled. Additional file 1: Figure S2 in the supplement the location of the participants distributed in the north-western suburbs of Adelaide.

### Statistical analysis

For categorical analysis of lung function by traffic density, cut points were based on tertiles. Participants were categorised into a high, medium and low vehicles density group (High: ≥15,270; Medium: 7180–15269; Low: ≤7179).

Mean lung function and FEV_1_ reversibility were calculated by density category. Regression analysis assessed the statistical significance in the differences in mean lung function relative to base level (the lowest vehicle density) including confounders. The results of this analysis are presented in Table 3. Further analysis using the continuous measure of traffic density as a predictor in a multiple regression analysis estimated the average changes in lung function associated with increases in the 24 h density of vehicles (increment by 1000 vehicles). An interaction term between COPD (yes/no) and the continuous density variable was used in the regression analysis. The results of this analysis are presented in Table 4. Confounders included were age, body mass index (kg/m^2^), [BMI was calculated as weight (kg)/height (m) squared] smoking (currently, ever smoked), education level, annual gross household income and Socio-Economic Indexes for Areas (SEIFA) index of relative socio-economic advantage and disadvantage (IRSAD), created by the Australian Bureau of Statistics [20]. Statistical analysis was conducted in Stata 13.1.

## Results

Clinic assessment in the second stage of the NWAHS cohort occurred in 3206 participants of whom 3,103 completed post-bronchodilator spirometry. COPD was identified in 221 participants (7.1 %). Table 1 presents the characteristics of participants by COPD and by vehicle density.

Participants with COPD were significantly older than those without COPD [mean (SD) age 68.2 (11.9) compared to 53.9 (15.3)]. Hence, the categorical age distribution by category of density was also different between the COPD and no COPD group, with a high percentage of people in the two older and less subjects in the younger age groups in those with COPD. Current and ever smoking prevalence was significantly higher, education and income levels were lower, in the COPD group compared to the non-COPD group. When proximity to a major road was assessed against vehicle density categories, it was clearly demonstrated that people living closest to major roads were also exposed to a greater vehicle density. This distribution was similar in the COPD and non-COPD category.

Table 2 compares characteristics and confounding variables in COPD subjects by gender. COPD was significantly more common in males [*n* = 150 (67.9 %)] compared to females [*n* = 71 (32.1 %)]. Compared to females, males with COPD were significantly more likely to have a smoking history, reside in closer proximity to main roads, and be more likely to have post-secondary school qualification.

**Table 2:** Characteristics of participants with COPD by gender (*n* = 221)

Characteristics	Female *N* = 71 (%)	Male *N* = 150 (%)
Age		
20–39	1.4	1.3
40–59	26.8	19.3
60+	71.8	79.3
Smoking status		
Current smoking	25.4	22.0
Ever smoked#	27.6	72.4
Education*		
Left school at ≤15	40.6	30.4
After 15	30.4	17.6
Trade/apprentice or Certificate/Diploma	21.7	49.3
Tertiary	7.3	2.7
Household Income		
≤ $20, 000	40.9	38.1
> $20, 000–$40, 000	36.4	42.5
> $40, 000–$60, 000	13.6	10.8
> $60, 000	9.1	8.6
SEIFA IRSAD quintiles		
Lowest-low quintile combined	60.6	59.1
Middle quintile	12.7	20.1
High and highest quintile combined	26.8	20.8
Proximity to major road# (>10, 000 vehicles per day)		
> 300 m	69.0	58.0
150–300 m	9.9	23.3
< 150 m	21.1	18.7

*P < 0.05; # < 0.1

*SEIFA* socio-economic indexes for areas index of relative socio-economic advantage and disadvantage (IRSAD)

### Categories of density and lung function

**Fig. 1:**
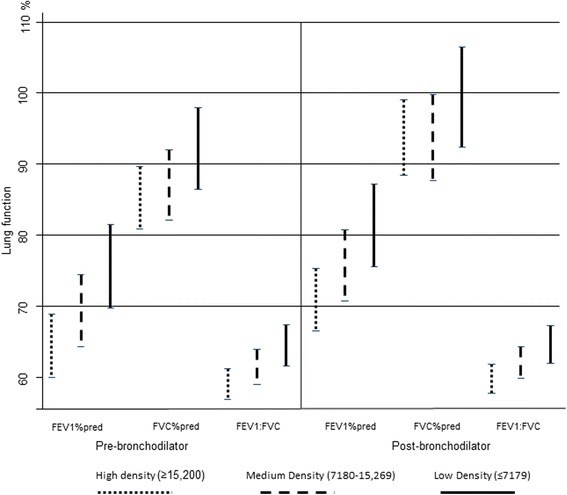
Adjusted mean lung function by category of density and 95 % confidence intervals in all COPD subjects

In those with COPD, mean lung function measures indicated an exposure-response relationship with increasing motor vehicle density for pre and post-bronchodilator measurements (Fig. 1).

Table 3 shows the adjusted mean (95 % confidence interval) post-bronchodilator lung function parameters (pre-bronchodilator data can be viewed in Additional file 1: Table S1 in the supplement) in relation to categories of vehicle density in participants with COPD. The respective results for those without COPD can be found in Additional file 1: Table S2 in the on-line supplement and no differences were observed in relation to vehicle density categories.

**Table 3:** Mean^a^ (95 % CI) post-bronchodilator lung function in subjects with COPD by vehicle density per 24 h within a 200 m buffer

	Post-bronchodilator [Mean (95 % CI)]			
	FEV_1_ % predicted	FVC % predicted	FEV_1_: FVC (%)	FEV_1_ reversibility (%)
All subjects (*n* = 221)				
low	81.4 (75.6–87.2)	99.5 (92.4–106.5)	64.6 (61.9–67.2)	8.4 (6.5–10.3)
medium	75.7 (70.7–80.7)	93.7 (87.7–99.8)	62.1 (59.8–64.3)	8.9 (7.1–10.7)
high	70.9* (66.5–75.3)	93.8 (88.4–99.1)	59.8* (57.8–61.8)	10.9 (8.9–13.0)
Males (*n* = 150)				
low	80.3 (73.6–87.0)	95.6 (89.6–101.7)	64.1 (60.6–67.7)	8.5 (6.3–10.8)
medium	78.0 (73.0–83.1)	94.4 (89.8–99.0)	62.2 (59.6–64.9)	8.8 (6.9–10.6)
high	71.9* (66.7–77.1)	93.2 (88.5–98.0)	58.9* (56.1–61.6)	10.6 (7.9–13.3)
Females (*n* = 71)				
low	80.5 (69.7–91.2)	103.8 (86.7–120.9)	64.6 (60.6–68.6)	8.2 (4.6–11.7)
medium	69.7 (55.8–83.6)	93.6 (71.5–115.7)	61.9 (56.7–67.0)	9.4 (4.5–14.3)
high	69.1# (61.4–76.8)	94.9 (82.7–107.2)	61.3 (58.5–64.2)	11.5 (8.3–14.6)

*p < 0.05; #*p* < 0.1, for high (≥15,269 vehicles) compared to low exposure (<7179 vehicles)

^a^Data are presented as adjusted means (95 % CI). Statistical significance between the low and high density group was tested using regression analysis including confounders (age, smoking habits, BMI, education level, annual household income and SEIFA Index of Relative Socio-Economic Disadvantage)

*FEV*_1_ forced expiratory volume in 1 s, *FVC* forced vital capacity

For all subjects with COPD and for males, % predicted FEV_1_ and the FEV_1_/FVC ratio were significantly lower in the highest vehicle exposure group compared to the low density group in the adjusted regression analysis. The difference in mean % predicted FEV_1_ between the low and high vehicle density category was 10.5 percentage points in all COPD participants and 8.4 percentage points in males. For the FEV_1_/FVC ratio, differences between the low and high vehicle density categories were 4.8 percentage points in all subjects and 5.2 in males respectively.

In females, the results were similar. A borderline significance was observed for post-bronchodilator % predicted FEV1 with a difference in percentage points between the low and high vehicle density category of 11.4 percentage points.

Post-bronchodilator reversibility showed no significant change with increasing vehicle density overall or by sex.

### Regression analysis of lung function and vehicle density

Table 4 shows the unadjusted and adjusted reductions in post-bronchodilator lung function parameters calculated for an increase in 1000 vehicles over 24 h within a 200 m buffer around residences of subjects with COPD (pre-bronchodilator data can be viewed in Additional file 1: Table S3 in the supplement). The point estimates of the lung function parameters indicated a general inverse relationship with density.

**Table 4:** Post-bronchodilator lung function estimates correlated with traffic density within a 200 m buffer for all COPD subjects and by gender

	Post-bronchodilator lung function		
	Unadjusted unstandardized coefficients (95 % CI)		
	FEV_1_ % predicted	FVC % predicted	FEV_1_: FVC
All subjects	−0.05* (−0.09,−0.01)	−0.01 (−0.05, 0.03)	−0.02* (−0.04,−0.01)
Males	−0.06* (−0.11,−0.01)	−0.03 (−0.07, 0.02)	−0.03* (−0.04,−0.01)
Females	−0.02 (−0.11, 0.07)	−0.04 (−0.04, 0.13)	−0.01 (−0.04, 0.02)
Adjusted unstandardized coefficients (95 % CI)			
All subjects	−0.05* (−0.09,−0.01)	−0.01 (−0.05, 0.04)	−0.03** (−0.04, 0.01)
Males	−0.06** (−0.11,−0.02)	−0.03 (−0.07, 0.01)	−0.03** (−0.05,−0.01)
Females	−0.003 (−0.01, 0.09)	−0.08# (−0.01, 0.17)	−0.02 (−0.05, 0.01)

Adjusted models included age, current smoking, highest qualification, income, BMI, and SEIFA Index of Relative Socio-Economic Advantage and Disadvantage (IRSAD). **p < 0.01 *p < 0.05; #*p* < 0.1

Coefficients represent the change in lung function for an increase per 1000 vehicles/24 h. Analysis was conducted using an interaction term between COPD (yes/no) and linear density of vehicles

In all subjects and in males, post-bronchodilator FEV1/FVC and % predicted FEV_1_, but not FVC % predicted were significantly reduced in association with increasing vehicle density, before and after adjustment for confounders. No significant linear relationship between lung function parameters and density was observed in females. When analyses were conducted with smoking considered as ‘ever smoked’ in addition to ‘current smoking’, the point estimates did not change and only the post-bronchodilator % predicted FEV_1_ in males changed to borderline significance.

We conducted a sensitivity analysis excluding the three highest car density observations, consistent with the 99th percentile. The linear regression results were robust to the removal of these observations.

## Discussion

This is, to our best knowledge, the first study to consider the effect of motor vehicle density on lung function parameters within an unselected COPD population. We hypothesised that lung function of people with COPD would be differentially affected according to where they live with regard to vehicle density, a proxy for air quality. Any findings of lung function reduction would have implications for the progression of COPD over time regardless of the multifactorial contributors which caused COPD.

Our study demonstrated a clear association between lung function parameters and density of vehicles reflecting a 24 h exposure in a 200 m buffer around participants with COPD residing in north-west regions of Adelaide that was stronger in men than women. The analysis by category of density showed significant reductions in lung function. For % predicted FEV_1_ for example, the difference from low to high vehicle density was 10 percentage points in males and 8 in females respectively. In the linear regression, the highest reduction was 0.07 % of predicted FEV_1_ per 1000 vehicles in male subjects. COPD patients exposed to the equivalent of the 50th road density percentile in this study would be exposed to 11,000 vehicles and those in the 90th percentile to 91,000 vehicles leading to an average reduction in percentage points of predicted FEV_1_ of 0.8 and 6.4 % respectively. Adelaide’s daily traffic volume on any single major road does not exceed 76,500 vehicles [21]. In contrast, many of the world’s city highways have a daily volume of more than 200,000 vehicles contributing to overall higher vehicle densities than observed in this study when accounting for the addition of vehicles from other road sections in the 200 m buffer density [11]. This may be associated with concomitant larger lung function detriments. Furthermore, we did not find a significant association between vehicle density and post-bronchodilator reversibility suggesting that traffic exposure may contribute to a fixed chronic obstruction of the airways rather than an acutely reversible obstruction leading to FEV1 decline.

There is no consensus on the spirometric definition of COPD. We used a fixed ratio of FEV1/FVC of <0.70 to identify COPD consistent with the GOLD guideline [14]. Evidence from large population cohorts shows that subjects classified as “normal” using the lower limit of normal (LLN), but abnormal with the fixed ratio, demonstrate an increased risk of death and COPD-related hospitalisation during follow-up compared with asymptomatic individuals with normal lung function [15, 17]. The use of LLN has recently been shown to fail to identify a number of patients with significant pulmonary pathology and respiratory morbidity compared to the fixed ratio [16]. In our study 69.8 % of participants identified by the fixed ratio were also identified by LLN criteria and the remaining 30.2 % (*N* = 68) also demonstrated a post-bronchodilator FEV_1_ of <80 % predicted, indicative of significant respiratory impairment [22].

Very few studies have used objective spirometric lung measurements in adults, and none have used them to discern relative decrements in lung function associated with traffic-related air pollution in samples of people with COPD. In general population samples, spirometry measures have been used recently to determine prevalence of COPD in relation to traffic exposures or surrogates with mixed results [8–10, 23, 24]. Collectively, these studies suggest that COPD may be associated with air pollution exposure [25]. Some of these studies reported lung function levels in association with traffic proxies in the general population and indicated reductions that were in magnitude comparable to our study. Kan et al. [8] reported adjusted FEV_1_ and FVC reductions related to traffic density which were significantly decreased across quartiles of density using the lowest density as comparison category. Nuvolone et al. [9] compared lung function parameters by distance to a busy road (as opposed in our study) and reported that in males, the reduction in FEV_1_/FVC ratio was 1.4 % when comparing participants living <100 m compared to >250 m distance from this roadway. However, the level of vehicle exposure was not reported in this study and a direct comparison to the difference in FEV_1_/FVC between the high and the low vehicle density category in our study in males of 5.5 % is not possible [9].

Lung function decline has been directly linked to the frequency of COPD exacerbations [26]. Episodes of worsening COPD have been frequently linked to general and traffic-related air pollution causing increased morbidity and mortality in COPD patients [6]. Air pollution levels in cities have been associated with an increase in daily COPD-related hospital admissions [27, 28] and mortality [29], as well as frequency of COPD exacerbations [30].

Fine particulate matter with aerodynamic diameter <2.5 μm (PM2.5) is derived from combustion processes that include emissions from motor vehicle use, industry, biomass burning and smoking. PM2.5 contains reactive oxygen species that induce inflammatory processes and cause overload in alveolar macrophages in the small airways causing inflammation due to release of various immune system-related cells and associated mediators [6, 11]. Combined evidence from epidemiological and toxicological research have concluded that COPD patients are particularly sensitive to PM2.5 [25, 31]. Toxicological studies have suggested that it is likely that repeated exacerbations of the respiratory system may cause permanent injury, higher susceptibility to infections and a re-modelling of airways even in the absence of smoking [32]. Continuous chronic exposure to PM2.5 in COPD patients is suggested to interact through a number of mechanisms with the already present COPD disease process [30, 31]. This can lead to exacerbations of COPD during high level of PM2.5 and over time to reductions in lung function relative to exposure status and ultimately to progression of COPD and related mortality and morbidity [6]. A study of women living in highly polluted areas demonstrated a statistically significant decline in lung function and increased COPD prevalence associated with a 5 year PM10 increase of 7.0 μg/m^3^ (interquartile range) [9]. The reductions in % predicted FEV1 and FVC were 5.1 % for and 3.7 % respectively. While PM has been previously associated with COPD, other co-pollutants including nitrogen dioxide and gaseous air toxics may also cause adverse effects, albeit, this evidence has not yet been developed.

One of the strengths of this study is that misclassification of COPD disease status is greatly reduced given objective clinical measurements [15]. Moreover, this study used post-bronchodilator spirometry values that international guidelines promote for use in the definition and severity classification of COPD [14]. Further points of strength were the population-based study design adding to the generalisability of the findings and the availability of relevant confounding variables. Smoking and socio-economic status-related variables (age, education, income) were considered to be the most important confounders in this cross-sectional study design, but others were also included (BMI, area level socioeconomic status). After adjustment the association between lung function parameters and vehicle density persisted.

There are a number of limitations in this study. The cross-sectional design precludes knowledge of the disease onset and progression over time. As the data from this study has been obtained from a cohort study, it will be possible to later analyse lung function changes over time and progress in air pollution modelling may improve accuracy of exposure classification. Further, we have no information on occupational lifetime exposure to air pollutants, a possible confounder considering some areas of blue collar workers in the study area, but it is likely that this information is taken into account by adjusting for level of socioeconomic status by area.

In this study, the categorical assessment in females showed significant lung function reductions by density and negative lung function point estimates for all outcomes in the linear regression analysis, but none of the latter reached statistical significance. The relatively small sample size of COPD patients is likely to have impeded a clear signal in the female lung function results. Two recent general population studies used traffic exposure proxies with gender-specific lung function findings. While Nuvolone et al. observed reductions in the FEV_1_/FVC ratio in males only, Kan observed FEV_1_ and FVC-related reductions in females only [8, 9]. These opposing results cannot be easily interpreted, but recent reviews on gender and COPD discuss common differential findings which may reflect the multifactorial development of COPD [33].

Density of motor vehicles only provides a proxy estimate of the exposure to fuel emissions. Modelled exposure measurements of PM2.5 or of other air pollutants are currently not available in SA, but the vehicle density, based on a 200 m buffer around subjects, was calculated at the individual level providing variability across the study population. The density metric may be associated with some random exposure misclassification with the effect of minimizing the association. It has been used successfully in a number of studies that established significant relationships with asthma health outcomes [34–36]. A study comparing effectiveness of exposure metrics for traffic-related air pollutants showed that density is a measure that is broadly in agreement with other more sophisticated proxy measures including road-based emissions and dispersion modelling [12].

The question is whether the results of this study can in any way benefit people with COPD. In the first instance, it is important for physicians and patients to know that traffic exposure may affect COPD. It may be possible to move to areas with less traffic and additional exposure to indoor air pollution through biomass combustion can be avoided. Studies in children and adults have demonstrated that improving air quality has a positive effect on lung function, symptoms and mortality [37–39]. These studies indicate a wide-reaching benefit from improved air quality not only for COPD patients, but for the whole population. A conventional smoking cessation intervention in smokers with mild to moderate COPD improved % predFEV_1_ by two percentage points in the first year among quitters [40]. This compares well in magnitude to an improvement of six to ten percentage points in % predFEV1 when reducing from high to low vehicle density as suggested in this study.

## Conclusion

Recent reviews have outlined a substantive role of air pollution in the short and long term progress of COPD. This study has demonstrated for the first time clinically important lung function reductions in relation to traffic density in people with spirometrically determined COPD. This has consequences for COPD patients considering that the difference between low density (<7179 vehicles within a 200 m diameter circle) and high density exposure (>15 269 vehicles) was associated with a reduction of up to 10 % points of FEV1 % predicted in the high density category. It is generally advised that COPD patients preserve their lung function by ceasing to smoke or to avoid occupational exposures. Traffic-related PM2.5 is similar to tobacco smoke-related or any combustion-related PM2.5, and all can potentially contribute to additional irreversible loss of pulmonary function, but it may be more difficult to suggest a change of residence to a less polluted area. Community-based interventions that improve air quality would assist in prevention of disease progression of COPD and would benefit the entire community considering that PM2.5 and other air pollutants are linked to other respiratory and to cardiovascular disease.

## Additional file

Additional file 1: Lung function reductions associated with motor vehicle density in chronic obstructive pulmonary disease: a cross-sectional study. (DOCX 968 kb)

## Abbreviations

BMI: Body mass index
COPD: Chronic obstructive bronchial disease
DALY: Global disability-adjusted life-year
EPA: Environment protection authority
FEV1: Forced expiratory volume in 1 s
FVC: Forced vital capacity
IRSAD: Index of relative socio-economic advantage and disadvantage
LLN: Lower limit of normal
NWAHS: North West Adelaide health study
PM: Particulate matter
PM10: PM with an aerodynamic diameter of less than 10 μm
PM2.5: PM with an aerodynamic diameter of less than 2.5 μm
SA: South Australia
Seifa: Socio-economic indexes for areas
