# Supplementary material for: Lung function reductions associated with motor vehicle density in chronic obstructive pulmonary disease: a cross-sectional study
Source: Respir Res. 2016 Oct 24;17:138. doi: 10.1186/s12931-016-0451-3 (PMC5078919; doi:10.1186/s12931-016-0451-3)
Supplement: Additional file 1: — Lung function reductions associated with motor vehicle density in chronic obstructive pulmonary disease: a cross-sectional study. (DOCX 968 kb) [file 12931_2016_451_MOESM1_ESM.docx]

Supplemental Material

**Lung function reductions associated with motor vehicle density in chronic obstructive pulmonary disease**

^1^ Monika Nitschke, ^2^Sarah L. Appleton, ^3^Qiaoyu Li, ^1^ Graeme R. Tucker, ^4^Pushan Shah, ^3^Peng Bi, ^3^Dino L. Pisaniello, ^2^Robert J. Adams.

1 Department for Health and Ageing, Adelaide, South Australia, Australia.

2 The Health Observatory, Discipline of Medicine, the University of Adelaide, Adelaide, South Australia, Australia

3 The School of Public Health, University of Adelaide, Adelaide, South Australia, Australia

4 Environment Protection Authority, Adelaide, South Australia, Australia

Corresponding Author: Dr Monika Nitschke, Department for Health and Ageing, 11 Hindmarsh Square, Adelaide, South Australia, email: [monika.nitschke@health.sa.gov.au](mailto:monika.nitschke@health.sa.gov.au)

Telephone: +61 8 82267126

Table of contents

Page 3 Figure S1: An example of a mapped geocode of a participant within a 200 m buffer.

Page 4 Figure S2: Location of all North West Adelaide Health Study participants in the North-West region of metropolitan Adelaide.

**Page 5** Table S1: Adjusted mean^†^ (95% CI) pre-bronchodilator lung function in subjects with COPD by vehicle density category per 24 hours within a 200 m buffer.

**Page 6** Table S2: Adjusted mean (95%CI) pre- and post-bronchodilator lung function in subjects without COPD by vehicle density per 24 hours within a 200 m buffer.

**Page 8** Table *S3:* Lung function estimates correlated with traffic density within a 200 m buffer for all COPD subjects and by gender.


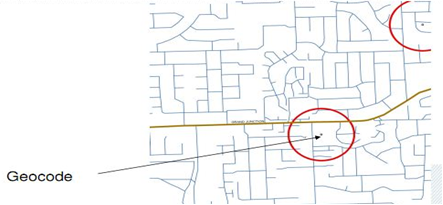


**Figure S1** An example of a mapped geocode of a participant within a 200 m buffer and the included road links from which the daily traffic count was calculated.


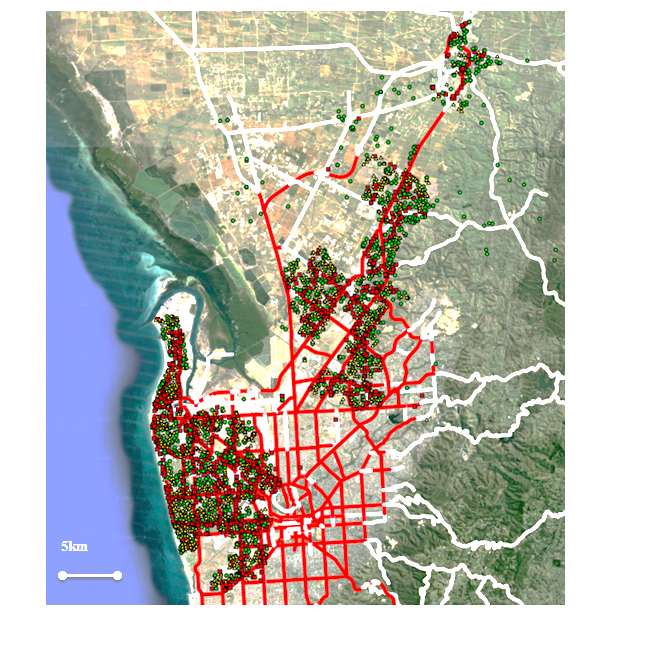


Figure S2 Location of all North West Adelaide Health Study participants in the North-West region of metropolitan Adelaide.

Table S1 Adjusted mean^†^ (95% CI) pre-bronchodilator lung function in subjects with COPD by vehicle density per 24 hours within a 200 m buffer

### *p<0.05; #p<0.1, for high (≥15,269 vehicles) compared to low exposure (<7179 vehicles).

^†^ Data are presented as adjusted means (95%CI). Statistical significance between the low and high density group was tested using regression analysis including confounders (age, smoking habits, BMI, education level, annual income and SEIFA Index of Relative Socio-Economic Disadvantage).

|  | Pre-bronchodilator lung function [Mean (95% CI)] | | |
| --- | --- | --- | --- |
|  | FEV_1_ % predicted | FVC % predicted | FEV_1_:FVC (%) |
| All subjects (n=221) |  |  |  |
| low | 75.6 (69.7-81.5) | 92.3 (86.5-98.0) | 64.5 (61.6-67.4) |
| medium | 69.40 (64.3-74.5) | 87.0 (82.1-92.0) | 61.5 (59.0-64.0) |
| high | 64.5* (60.0-68.9) | 85.3# (80.9-89.7) | 59.0* (56.8-61.2) |
| Males (n=150) |  |  |  |
| low | 75.0 (68.3-81.7) | 88.9 (82.7-95.2) | 64.3 (60.5-68.1) |
| medium | 71.9 (66.8-76.9) | 88.7 (83.9-93.4) | 61.3 (58.4-64.2) |
| high | 65.5*(60.3-70.7) | 87.0 (82.2-91.9) | 57.3* (54.4-60.3) |
| Females (n=71) |  |  |  |
| low | 74.5 (63.0-85.9) | 95.6 (84.0-107.3) | 64.7 (60.1-69.4) |
| medium | 61.4 (46.6-76.2) | 83.3 (68.2-98.4) | 61.8 (55.8-67.8) |
| high | 62.7* (54.5-70.9) | 82.6# (74.3-91.0) | 61.9 (58.6-65.3) |

Table S2 Adjusted mean (95%CI) pre- and post-bronchodilator lung function in subjects without COPD by vehicle density per 24 hours within a 200 m buffer. *p<0.05; #p<0.1.

Confounders included were age, smoking habits, BMI, education level, annual income and SEIFA index of relative socio-economic advantage and disadvantage

|  | **Pre-bronchodilator lung function [Mean (95% CI)]** | | |  |
| --- | --- | --- | --- | --- |
|  |  |  |  |  |
| Non-COPD | FEV_1_% predicted | FVC% predicted | FEV_1_:FVC | FEV_1_ reversibility (%) |
| **All subjects (n=2881)** |  |  |  |  |
| Low | 99.0 (97.8-100.2) | 100.6 (99.4-101.7) | 81.0 (80.5-81.4) |  |
| Medium | 81.4 (98.0-100.2) | 100.2(99.1-101.3) | 81.4 (81.0-81.6) |  |
| High | 98.9 (97.9-99.9) | 100.0 (00.1-100.9) | 81.3 (81.0-81.7) |  |
| **Males**  **(n=1334)** |  |  |  |  |
| Low | 97.1 (95.4-98.8) | 96.3 (94.7-97.9) | 80.2 (79.6-80.8) |  |
| Medium | 97.1 (95.5-98.6) | 95.8 (94.3-97.2) | 80.8 (80.3-81.4) |  |
| High | 96.1 (94.8-97.5) | 95.3 (94.1-96.6) | 80.3 (79.8-80.8) |  |
| **Females**  **(n=1547)** |  |  |  |  |
| Low | 100.7 (99.1-102.3) | 104.3 (102.8-105.9) | 81.7 (81.0-82.1) |  |
| Medium | 100.9 (99.3-101.7) | 103.9 (102.4-105.3) | 81.9 (81.4-82.5) |  |
| High | 101.2 (99.9-102.5) | 103.9 (102.7-105.2) | 82.3 (81.8-82.7) |  |
|  | **Post-bronchodilator lung function [Mean (95% CI)]** | | |  |
| **All subjects** |  |  |  |  |
| Low | 102.7 (101.5-103.8) | 100.9 (99.7-102.0) | 83.7 (83.3-84.1) | 3.9 (3.6-4.1) |
| Medium | 102.4 (101.3-103.5) | 100.7 (99.6-101.7) | 83.7 (83.4-84.1) | 3.7 (3.5-4.0) |
| High | 102.4 (101.5-103.3) | 100.6 (99.7-101.5) | 83.7 (83.4-84.0) | 3.9 (3.7-4.1) |
| **Males** |  |  |  |  |
| Low | 100.6 (99.0-102.3) | 96.7 (95.2-98.3) | 82.8 (82.2-83.4) | 3.9 (3.5-4.2) |
| Medium | 100.2 (98.7-101.3) | 96.2 (94.8-97.7) | 83.0 (82.5-83.6) | 3.7 (3.4-4.2) |
| High | 99.5 (98.2-100.9) | 95.9 (94.7-97.1) | 82.6 (82.2-83.1) | 3.9 (3.6-4.3) |
| **Females** |  |  |  |  |
| Low | 104.4 (102.8-106.0) | 104.6 (103.1-106.1) | 84.4 (83.9-84.9) | 3.8 (3.5-4.2) |
| Medium | 104.3 (102.8-105.8) | 104.4 (103.0-105.8) | 84.3 (83.8-84.8) | 3.8 (3.4-4.2) |
| High | 104.5 (103.3-105.7) | 104.5 (103.3-105.7) | 84.7 (84.3-85.1) | 3.9 (3.6-4.3) |

.

Table S3 Lung function estimates correlated with traffic density within a 200 m buffer for all COPD subjects and by gender. Coefficients represent the change in lung function for an increase per 1000 vehicles/24 hours. Analysis was conducted using an interaction term between COPD (yes/no) and linear density of vehicles. Adjusted models included age, current smoking, highest qualification, income, BMI, and SEIFA Index of Relative Socio-Economic Advantage and Disadvantage (IRSAD). **p<0.01 * p<0.05; #p<0.1

|  | Pre-bronchodilator lung function | | |
| --- | --- | --- | --- |
|  | FEV_1_ % predicted | FVC % predicted | FEV_1_/FVC (%) |
|  | Unadjusted unstandardized coefficients (95% CI) | | |
| All subjects | -0.05* (-0.10, -0.01) | -0.03 (-0.08;0.01) | -0.02* (-0.04, -0.01) |
| Males | -0.06* (-0.11, -0.01) | -0.03 (-0.07;0.02) | -0.03** (-0.05, -0.01) |
| Females | -0.03 (-0.1, 0.06) | -0.06 (-0.2;0.03) | -0.01 (-0.02, 0.04) |
|  | Adjusted unstandardized coefficients (95% CI) | | |
| All subjects | -0.05* (-0.10, -0.01) | -0.03 (-0.07, 0.01) | -0.03* (-0.04, -0.01) |
| Males | -0.07** (-0.11, -0.02) | -0.03 (-0.07, 0.02) | -0.03** (-0.05, -0.02) |
| Females | -0.02 (-0.11, 0.08) | -0.03 (-0.12, 0.06) | -0.01 (-0.03, 0.03) |
